# Immune reconstitution and long-term survival outcomes in LTHIs and TPs living with long-term ART: a propensity score-matched cohort study in Xinjiang, China

**DOI:** 10.3389/fpubh.2026.1845386

**Published:** 2026-08-05

**Authors:** Zikang Hu, Yongkang Ni, Qian He, Fengying Wang, Zhen Ni, Xiaoyan He, Xinhuan Feng, Mingjian Ni

**Affiliations:** 1 School of Public Health, Xinjiang Medical University, Ürümqi, China; 2 STD/HIV Prevention and Control Center, Xinjiang Uygur Autonomous Region Centre for Disease Control and Prevention, Ürümqi, China; 3 Yining Second People's Hospital, Yining, China; 4 Yining People's Hospital, Yining, China

**Keywords:** immune reconstitution, long-term HIV-1 infected individuals, propensity score matching, survival analysis, typical progressors

## Abstract

**Background:**

To compare immune reconstitution and long-term survival outcomes between long-term HIV-1 infected individuals (LTHIs) and typical progressors (TPs) under long-term antiretroviral therapy (ART) using a prospective HIV cohort in Yining City, China, and to provide evidence for risk stratification and follow-up management in long-term HIV care.

**Methods:**

The study included eligible people living with HIV (PLWH) from a prospective cohort in Yining City, China, with the observation period ending on 1 April 2024. Immune reconstitution (IR) was defined as the most recent CD4^+^ T-cell count ≥350 cells/μL before the data cut-off. Propensity score matching (PSM) was used to balance baseline differences between groups. Poisson regression with robust variance was applied to estimate relative risks (RR) and 95% confidence intervals (95% CI) for IR, and absolute risk differences (RD) were also calculated. Stratified analyses were performed by sex and age. Kaplan–Meier curves, log-rank tests, and Cox proportional hazards models were used to compare all-cause mortality.

**Results:**

A total of 4,844 PLWH were included, comprising 2,788 LTHIs and 2,056 TPs. After PSM, 1,799 matched pairs were obtained. In the matched cohort, IR was achieved in 76.9% (1,384/1799) of LTHIs and 35.9% (646/1799) of TPs. LTHIs had a markedly higher probability of IR, with an absolute risk difference of 41.0% and an adjusted RR of 2.04 (95% CI: 1.92–2.17; *p* < 0.001). Stratified analyses showed generally consistent findings across sex and age subgroups. For time-to-event analyses, 1,292 LTHIs and 1,757 TPs had valid post-classification follow-up time. During follow-up, 140 deaths occurred among LTHIs and 557 among TPs. Kaplan–Meier curves showed significantly better cumulative survival in LTHIs than in TPs (log-rank *p* < 0.001). In the adjusted Cox model, LTHIs had a significantly lower risk of all-cause mortality than TPs (adjusted HR = 0.50, 95% CI: 0.41–0.60; *p* < 0.001).

**Conclusion:**

Even under long-term ART, LTHIs showed a higher probability of immune reconstitution and lower post-classification all-cause mortality compared with TPs. These findings suggest that the initial disease progression pattern was independently associated with immune reconstitution and post-classification mortality risk, and may help inform risk stratification in long-term HIV care.

## 1 Introduction

In the era of combination antiretroviral therapy (ART), sustained virological suppression has become an achievable goal for most people living with HIV (PLWH), and earlier ART initiation has markedly reduced severe HIV-related illness and death (1–3). HIV infection is increasingly managed as a chronic condition rather than a rapidly fatal disease (4). Nevertheless, virological control does not necessarily translate into uniform immune recovery. Even among individuals with suppressed plasma HIV RNA, a substantial proportion fail to achieve adequate CD4^+^ T-cell restoration after ART (5, 6). This phenomenon, often described as immunological non-response or discordant immune response, remains clinically important because affected patients continue to show persistent immune dysfunction despite treatment (7, 8).

Incomplete immune recovery has been linked to worse long-term outcomes. Studies have shown that individuals with poor CD4 recovery during durable viral suppression remain at increased risk of long-term mortality (9, 10). In addition, persistent immune imbalance, reflected for example by a low CD4/CD8 ratio, has been associated with a higher risk of serious non-AIDS events and death in treated populations (11–13). Such findings suggest that virological suppression alone does not fully capture long-term prognosis. Even under contemporary care, PLWH may still experience a reduced life expectancy free of major chronic comorbidities compared with the general population (14).

HIV progression is highly heterogeneous across individuals. Previous studies have mainly characterized this heterogeneity through rare untreated phenotypes, such as long-term non-progressors (LTNPs) (15–17) and elite controllers (18), which have provided important insights into delayed disease progression and natural viral control. In China, previous studies have described the epidemiological features of LTNPs (19, 20) and identified factors associated with progression to AIDS or death after HIV diagnosis (21–23). However, these studies do not fully answer a clinically important question in the ART era: whether the initial pattern of HIV disease progression remains associated with immune reconstitution and long-term survival after PLWH have entered long-term ART care. In particular, direct comparisons between ART-treated long-term HIV-1-infected individuals (LTHIs), who remain clinically stable beyond the average latency period, and typical progressors (TPs), who develop AIDS-related manifestations or severe immunological deterioration within this period, remain limited, especially in Chinese cohorts with extended follow-up. Although TPs are expected to have more advanced disease status at or before treatment initiation, the incremental clinical value of directly comparing these two populations lies in determining whether the early progression phenotype continues to distinguish long-term immune and survival outcomes under ART. Such a comparison may help clarify whether disease progression patterns can serve as a practical marker for risk stratification, follow-up intensity, immune monitoring, and long-term management in treated populations. Therefore, using an ongoing prospective HIV cohort in Yining City, China, the present study compared immune reconstitution and long-term survival between LTHIs and TPs receiving long-term ART after propensity score matching.

## 2 Materials and methods

### 2.1 Study population and design

This study was conducted within an ongoing prospective HIV cohort in Yining City, Xinjiang Uyghur Autonomous Region, China. PLWH were entered into the cohort at the time of diagnosis and subsequently followed through a standardized management system. The cohort systematically recorded sociodemographic data, HIV-related clinical characteristics, treatment information, and laboratory results. By 1 April 2024, 7,508 PLWH had been registered in the cohort.

Long-term HIV-1 infected individuals (LTHIs) were defined as PLWH who fulfilled all of the following criteria: (1) adults diagnosed with HIV infection at a medical institution who voluntarily agreed to participate in the study; (2) a history of ART; (3) no AIDS-stage-related clinical symptoms or signs; and (4) a disease duration exceeding the average latency period of HIV infection (7.5 years from HIV diagnosis). Typical progressors (TPs) were defined as PLWH who met the following criteria: (1) adults diagnosed with HIV infection at a medical institution who voluntarily agreed to participate in the study; (2) development of AIDS-related symptoms or signs within the average latency period of HIV infection before initiation of first-line ART. Participants in both groups were excluded if they had: (1) opportunistic infections or AIDS-related malignancies at enrollment; (2) severe concomitant cardiovascular, respiratory, or hematological diseases, or other malignancies; (3) an inability to provide informed consent.

Written informed consent was obtained from all participants, and the study protocol was approved by the institutional ethics committee.

We followed the Strengthening the Reporting of Observational Studies in Epidemiology (STROBE) reporting guidelines throughout the investigation.

### 2.2 Data collection

HIV-related information was obtained from the cohort’s standardized data collection system. Variables collected included sociodemographic characteristics, such as age, sex, and marital status; route of HIV infection; previous ART regimens; prior use of trimethoprim-sulfamethoxazole prophylaxis; dates of HIV diagnosis and ART initiation; and serial viral load (VL) and CD4 cell count measurements. The relevant time points were defined using routinely collected cohort records. The HIV diagnosis date was defined as the first recorded date of confirmed HIV antibody positivity. The ART initiation date was defined as the recorded start date of first-line antiretroviral therapy. Group assignment was determined retrospectively according to predefined phenotype criteria based on available clinical and laboratory records. For LTHIs, phenotype classification was considered to occur 7.5 years after HIV diagnosis, provided that no AIDS-stage-related clinical symptoms or signs had been recorded before that time. For TPs, phenotype classification was considered to occur at the date of the first available CD4^+^ T-cell measurement used to identify typical progression status.

### 2.3 Outcome definitions

#### 2.3.1 Immune reconstitution

The primary outcome was immune reconstitution (IR). IR status was determined using the most recent CD4^+^ T-cell count recorded before the data cut-off (April 1, 2024). Participants were classified as having achieved IR if the most recent CD4^+^ T-cell count was ≥350 cells/μL, and as not having achieved IR if it was <350 cells/μL. This threshold was selected because a CD4^+^ T-cell count of 350 cells/μL falls within the range previously used to define immunological non-response after effective ART, and therefore provides a clinically interpretable threshold for assessing immune recovery in long-term treated PLWH. In this study, “most recent CD4” and “most recent VL” referred to the latest available laboratory results recorded before the data cut-off.

#### 2.3.2 Survival outcome

To evaluate long-term clinical outcomes, all-cause mortality was analyzed as the time-to-event outcome. For the survival analysis, follow-up started from the date of phenotype classification. The event time was defined as the date of death. Individuals who had not died by the end of follow-up were censored at the date of their last valid follow-up record or at the cut-off date, whichever came first. Individuals without positive follow-up time were excluded from time-to-event analyses. Accordingly, the survival analysis focused on post-classification all-cause mortality risk among participants who had already met the predefined criteria for LTHI or TP status.

### 2.4 Statistical analysis

All statistical analyses were performed in R statistical software (version 4.3.3). Continuous variables were presented as medians with interquartile ranges (IQR), and categorical variables were described as frequencies and proportions. Between-group comparisons were performed using the Wilcoxon rank-sum test for continuous variables, and the chi-square test or Fisher’s exact test for categorical variables, as appropriate. All statistical tests were two-sided, with *p* < 0.05 considered statistically significant.

Propensity score matching (PSM) was used to balance baseline differences between LTHIs and TPs. Propensity scores were estimated using a multivariable logistic regression model with group assignment as the dependent variable. Covariates included age, sex, marital status, most likely route of HIV acquisition, ART duration, and baseline VL category. These covariates were obtained from routinely collected records available before or at phenotype classification. One-to-one nearest-neighbor matching without replacement was then performed using a caliper width of 0.05 of the standard deviation of the logit of the propensity score. Covariate balance before and after matching was assessed using the standardized mean difference (SMD), with SMD < 0.10 considered indicative of adequate balance. After matching, 1,799 participants were retained in each group.

In the matched cohort, the probability of IR was compared between groups. Relative risks (RR) and 95% confidence intervals (CI) were estimated using Poisson regression with robust variance, and absolute risk difference (ARD) was also calculated.

For all-cause mortality, Kaplan–Meier curves with log-rank tests were used to compare survival between groups. Cox proportional hazards regression models were further used to estimate hazard ratios (HR) and 95% CIs for all-cause mortality, with robust standard errors clustered by matched pairs. The proportional hazards assumption was assessed using Schoenfeld residuals; if violated, extended Cox models with time-group interaction terms were fitted.

Prespecified subgroup analyses were conducted by sex, age group, route of transmission, WHO clinical stage, and baseline VL category, and the corresponding HRs with 95% CIs were presented in forest plots. For consistency, TPs were used as the reference group in all regression models and subgroup analyses. Therefore, relative risks, hazard ratios, and absolute risk differences were interpreted as estimates for LTHIs compared with TPs unless otherwise specified.

## 3 Results

### 3.1 Baseline characteristics before and after matching

**Figure 1:**
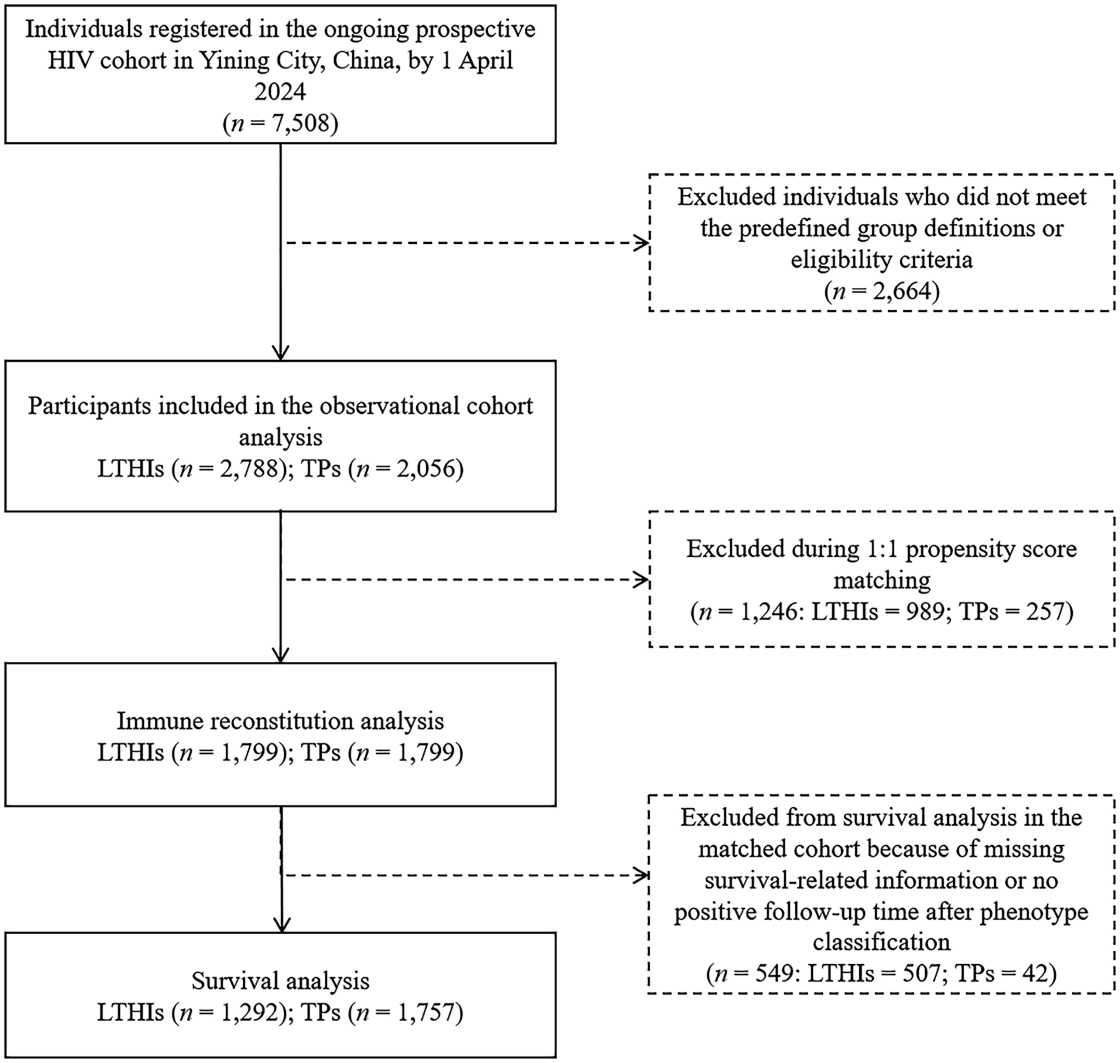
Flowchart of participant selection, propensity score matching, and inclusion in immune reconstitution and survival analyses.

After screening, 4,844 eligible PLWH were included, comprising 2,788 LTHIs and 2,056 TPs (Figure 1). Propensity scores were estimated using sociodemographic characteristics, route of HIV transmission, ART duration, and baseline VL category, and 1:1 nearest-neighbor matching yielded a matched cohort of 1,799 LTHIs and 1,799 TPs. As shown in Table 1, covariate balance improved substantially after matching, with most SMDs below 0.10 and only a small residual imbalance in the age-category distribution (SMD = 0.117). In the matched cohort, the median age was 37.4 years (IQR: 32.2–43.9) among LTHIs and 37.6 years (IQR: 33.0–43.5) among TPs, and men accounted for 62.20 and 60.37% of the two groups, respectively. Heterosexual transmission was the predominant route of HIV acquisition in both groups, followed by injecting drug use. Baseline virological status was also broadly comparable after matching; the proportions with plasma HIV VL < 20 copies/mL were 34.46% in LTHIs and 32.18% in TPs. The distributions of baseline laboratory indices are also presented in Table 1.

**Table 1:** Baseline clinical characteristics of LTHIs and TPs before and after matching.

Baseline characteristics	Cohorts before matching (*n* = 4,844)			Matched cohorts (*n* = 3,598)		
	LTHIs	TPs	SMD	LTHIs	TPs	SMD
Sample size, *n* (%)	2,788 (100%)	2056 (100%)	NA	1799 (100%)	1799 (100%)	NA
Sociodemographic characteristics						
Age (years), *n* (%)						
18–25	142 (5.13%)	53 (2.59%)	0.142	78 (4.36%)	50 (2.80%)	0.117
25–50	2,401 (86.74%)	1791 (87.67%)		1,535 (85.90%)	1,583 (88.63%)	
50–65	191 (6.90%)	174 (8.52%)		148 (8.28%)	141 (7.89%)	
≥ 65	34 (1.23%)	25 (1.22%)		26 (1.45%)	12 (0.67%)	
Median age [IQR]	36.7 [31.3, 42.8]	38.0 [33.4, 43.8]	0.158	37.4 [32.2, 43.9]	37.6 [33.0, 43.5]	0.010
Sex, *n* (%)						
Male	1,574 (56.46%)	1,309 (63.67%)	0.148	1,119 (62.20%)	1,086 (60.37%)	0.038
Female	1,214 (43.54%)	747 (36.33%)		680 (37.80%)	713 (39.63%)	
Marital status, *n* (%)						
Single	335 (12.02%)	263 (12.79%)	0.031	74 (10.12%)	79 (10.81%)	0.049
Married or living with partners	1722 (61.76%)	1,254 (60.99%)		440 (60.19%)	436 (59.64%)	
Divorced or widowed	723 (25.93%)	535 (26.02%)		194 (26.54%)	191 (26.13%)	
Unknown	8 (0.29%)	4 (0.19%)		23 (3.15%)	25 (3.42%)	
Route of HIV transmission, *n* (%)						
Injecting drug use	853 (30.60%)	771 (37.50%)	0.160	249 (34.06%)	237 (32.42%)	0.019
Heterosexual transmission	1,315 (47.17%)	826 (40.18%)		296 (40.49%)	299 (40.90%)	
Homosexual transmission	52 (1.87%)	36 (1.75%)		12 (1.64%)	10 (1.37%)	
Others	568 (20.37%)	423 (20.57%)		172 (23.53%)	182 (24.90%)	
Unknown	8 (0.30%)	5 (0.33%)		2 (0.27%)	2 (0.27%)	
ART duration, years [IQR]	11.0 [8.9, 12.3]	11.8 [8.7, 14.6]	0.233	11.1 [9.3, 12.3]	11.6 [7.9, 13.9]	0.032
Baseline laboratory test results, *n* (%)						
Plasma HIV VL (copies/mL), *n* (%)						
<20	1,312 (47.06%)	582 (28.31%)	0.402	620 (34.46%)	579 (32.18%)	0.050
20–200	535 (19.19%)	583 (28.36%)		446 (24.79%)	456 (25.35%)	
200–400	71 (2.55%)	70 (3.40%)		55 (3.06%)	57 (3.17%)	
400–1,000	137 (4.91%)	105 (5.11%)		88 (4.89%)	95 (5.28%)	
≥1,000	733 (26.29%)	716 (34.82%)		590 (32.80%)	612 (34.02%)	

### 3.2 Immune reconstitution in the matched cohort

IR occurred in 76.9% of LTHIs compared with 35.9% of TPs in the matched cohort. Using TPs as the reference group, LTHIs had a markedly higher probability of achieving immune reconstitution, corresponding to an absolute risk difference of 41.0 percentage points. The crude RR was 2.13 (95% CI: 2.00–2.27), and this association remained evident after adjustment (adjusted RR = 2.04, 95% CI: 1.92–2.17), indicating that LTHIs had substantially better immune recovery than TPs even after baseline balancing.

### 3.3 Subgroup analyses of immune reconstitution

Subgroup analyses showed that the higher probability of immune reconstitution among LTHIs, compared with TPs, was generally consistent across sex and age strata (Table 2). Among women, immune reconstitution occurred in 84.0% of LTHIs and 46.6% of TPs, corresponding to an absolute risk difference of 37.4 percentage points and an adjusted RR of 1.75 (95% CI: 1.59–1.89). Among men, the corresponding proportions were 72.7 and 28.9%, with a larger absolute risk difference of 43.7 percentage points and an adjusted RR of 2.38 (95% CI: 2.17–2.63). Thus, the immune recovery advantage of LTHIs was observed in both sexes and appeared more pronounced in male participants.

**Table 2:** Subgroup analysis of absolute risk differences and relative risks for immune reconstitution between LTHIs and TPs in the matched cohort.

Stratification	Sample size	Event at follow-up *n*,(%)	ARD	RR (95% CI)	
				crude	adjusted^$^
Overall					
LTHIs	1799	1,384 (76.9%)	41.00%	2.13 (2.00–2.27)	2.13 (2.00–2.27)
TPs	1799	646 (35.9%)	0.00%	1.00	1.00
Among female subjects					
LTHIs	680	571 (84.0%)	37.40%	1.82 (1.67–1.96)	1.82 (1.67–1.96)
TPs	713	332 (46.6%)	0.00%	1.00	1.00
Among male subjects					
LTHIs	1,119	813 (72.7%)	43.70%	2.50 (2.27–2.78)	2.50 (2.27–2.78)
TPs	1,086	314 (28.9%)	0.00%	1.00	1.00
Among subjects with age < 40 years					
LTHIs	1,090	823 (75.5%)	37.50%	2.00 (1.82–2.17)	2.00 (1.82–2.17)
TPs	1,117	424 (38.0%)	0.00%	1.00	1.00
Among subjects with age ≥ 40 years					
LTHIs	709	561 (79.1%)	46.60%	2.44 (2.17–2.70)	2.44 (2.17–2.70)
TPs	682	222 (32.6%)	0.00%	1.00	1.00

A similar pattern was observed across age strata. In participants aged <40 years, immune reconstitution occurred in 75.5% of LTHIs and 38.0% of TPs, yielding an absolute risk difference of 37.5 percentage points and an adjusted RR of 1.89 (95% CI: 1.75–2.08). In those aged ≥40 years, the corresponding proportions were 79.1 and 32.6%, with an even larger absolute risk difference of 46.6 percentage points and an adjusted RR of 2.27 (95% CI: 2.04–2.56). These findings indicate that the immune recovery advantage of LTHIs persisted across age groups and remained substantial in older individuals.

### 3.4 Long-term survival outcomes

**Figure 2:**
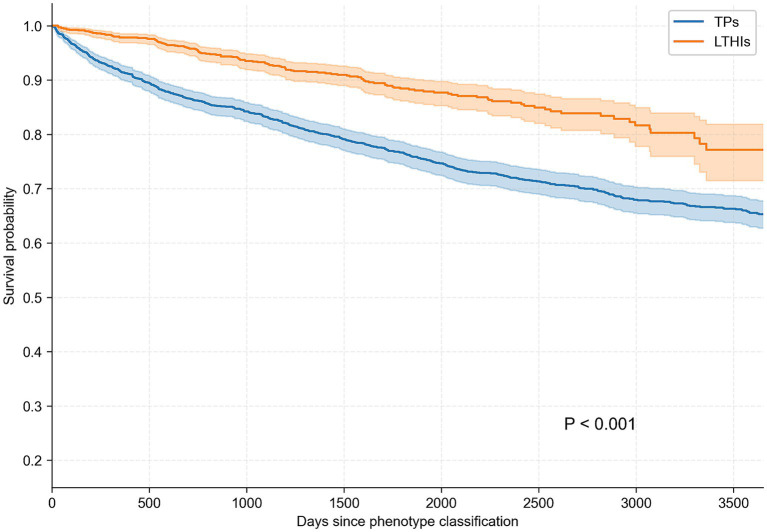
Kaplan–Meier survival curves for LTHIs and TPs after propensity score matching.

After redefining the start of follow-up as the phenotype classification date, 1,292 LTHIs and 1,757 TPs had valid post-classification follow-up time and were included in the revised survival analysis. In this survival-analysis subset, we further assessed covariate balance between groups. Most key propensity score matching covariates remained balanced, including sex, age at ART initiation, marital status, and route of HIV acquisition, with SMDs below 0.10. However, mild residual imbalance was observed for ART start year and baseline VL category, with SMDs of 0.133 and 0.125. Therefore, these variables were retained in the adjusted Cox regression model. During follow-up after phenotype classification, 140 deaths occurred among LTHIs, and 557 deaths occurred among TPs. Kaplan–Meier survival curves showed that LTHIs maintained a higher cumulative survival probability than TPs throughout follow-up after phenotype classification (Figure 2). The log-rank test indicated a statistically significant between-group difference (*p* < 0.001).

Using TPs as the reference group, the crude Cox model showed that LTHIs had a significantly lower risk of all-cause mortality than TPs (HR = 0.47, 95% CI: 0.39–0.56; *p* < 0.001). This association remained significant after adjustment for sex, age at ART initiation, marital status, route of transmission, ART start year, baseline viral load, and WHO clinical stage. In the adjusted model, LTHIs had approximately 50% lower all-cause mortality risk than TPs (adjusted HR = 0.50, 95% CI: 0.41–0.60; *p* < 0.001).

### 3.5 Subgroup analyses of long-term survival

**Figure 3:**
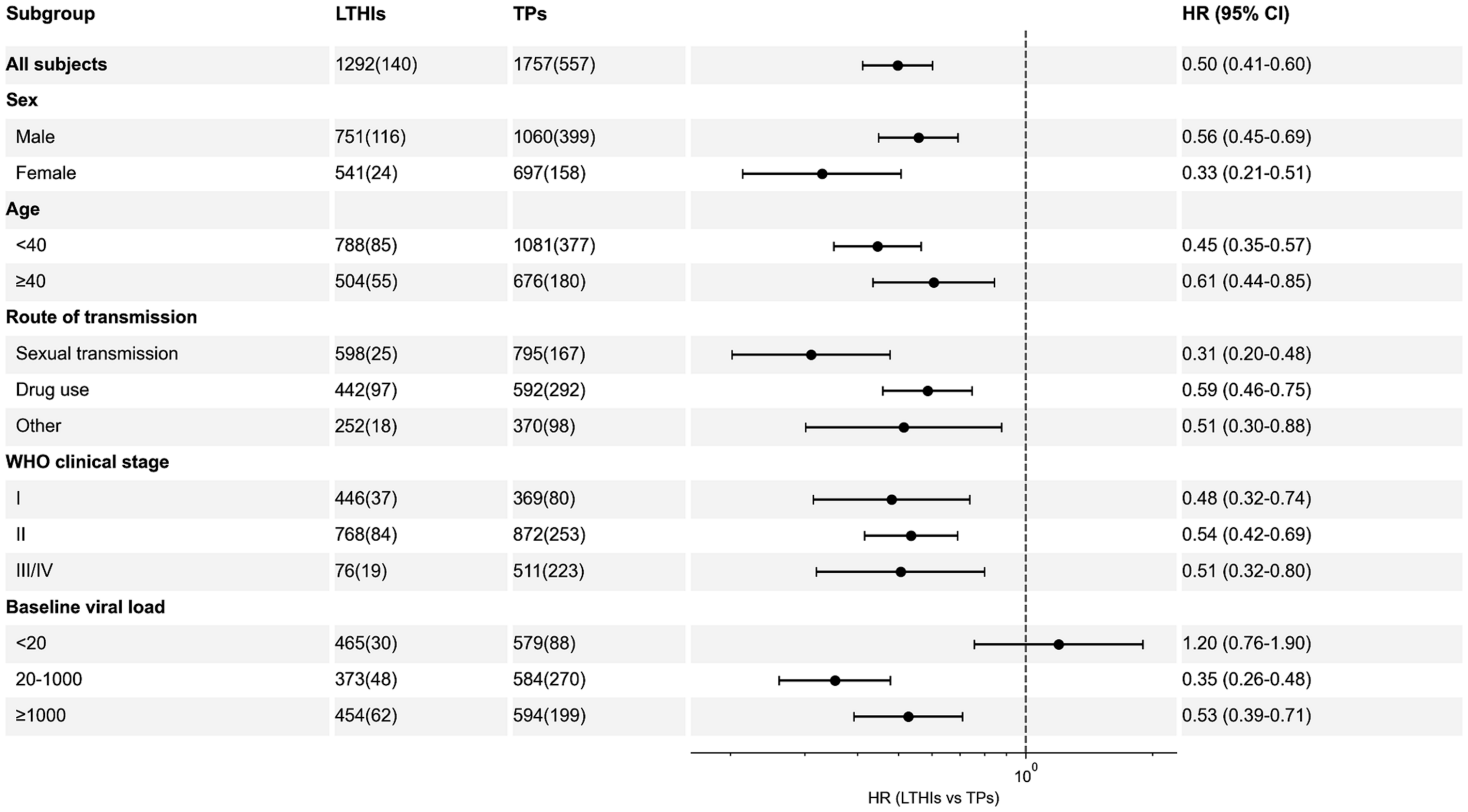
Subgroup analysis of all-cause mortality in the matched cohort.

Subgroup analyses showed that the association between LTHI status and lower all-cause mortality was generally consistent across clinically relevant strata (Table 3 and Figure 3). In sex-stratified analyses, the adjusted HRs for LTHIs versus TPs were 0.56 (95% CI: 0.45–0.69; *p* < 0.001) among men and 0.33 (95% CI: 0.21–0.51; *p* < 0.001) among women. Similar protective associations were observed in both age groups, with adjusted HRs of 0.45 (95% CI: 0.35–0.57; *p* < 0.001) among participants aged <40 years and 0.61 (95% CI: 0.44–0.85; *p* = 0.003) among those aged ≥40 years.

**Table 3:** Subgroup analysis of hazard ratios for all-cause mortality between LTHIs and TPs in the matched cohort.

Subgroup	LTHIs	TPs	HR (95% CI)	*P*
Overall	1,292 (140)	1757 (557)	0.50 (0.41–0.60)	<0.001
Sex				
Male	751 (116)	1,060 (399)	0.56 (0.45–0.69)	<0.001
Female	541 (24)	697 (158)	0.33 (0.21–0.51)	<0.001
Age (years), *n*				
<40	788 (85)	1,081 (377)	0.45 (0.35–0.57)	<0.001
≥40	504 (55)	676 (180)	0.61 (0.44–0.85)	0.003
Route of transmission, *n* (%)				
Sexual transmission	598 (25)	795 (167)	0.31 (0.20–0.48)	<0.001
Drug use	442 (97)	592 (292)	0.59 (0.46–0.75)	<0.001
Other	252 (18)	370 (98)	0.51 (0.30–0.88)	0.015
WHO clinical stage, *n* (%)				
I	446 (37)	369 (80)	0.48 (0.32–0.74)	<0.001
II	768 (84)	872 (253)	0.54 (0.42–0.69)	<0.001
III/IV	76 (19)	511 (223)	0.51 (0.32–0.80)	0.004
Plasma HIV VL (copies/mL), *n* (%)				
<20	465 (30)	579 (88)	1.20 (0.76–1.90)	0.440
20–1,000	373 (48)	584 (270)	0.35 (0.26–0.48)	<0.001
≥1,000	454 (62)	594 (199)	0.53 (0.39–0.71)	<0.001

The survival advantage of LTHIs was also observed across strata defined by route of transmission and WHO clinical stage. The adjusted HRs were 0.31 (95% CI: 0.20–0.48; *p* < 0.001) among participants with sexual transmission, 0.59 (95% CI: 0.46–0.75; *p* < 0.001) among those with drug use, and 0.51 (95% CI: 0.30–0.88; *p* = 0.015) among those with other transmission routes. Across WHO clinical stages I, II, and III/IV, the adjusted HRs were 0.48, 0.54, and 0.51, respectively. In analyses stratified by baseline viral load, the association was significant among participants with baseline viral load of 20–1,000 copies/mL and ≥1,000 copies/mL, whereas the estimate was not statistically significant among those with baseline viral load <20 copies/mL (HR = 1.20, 95% CI: 0.76–1.90; *p* = 0.440).

## 4 Discussion

In the ART era, sustained virological suppression has become an achievable goal for most PLWH, but viral suppression does not necessarily translate into complete immune recovery or uniformly favorable long-term outcomes. In the present study, based on a long-term prospective cohort in Yining City, we found that LTHIs had substantially better immune reconstitution and more favorable long-term survival than TPs. The difference in immune reconstitution was directionally consistent across sex and age subgroups, and the survival analyses further showed that LTHIs maintained a persistently higher probability of survival during follow-up. These findings suggest that differences in disease progression patterns remain clinically relevant even under long-term ART and may continue to shape both immunological recovery and long-term prognosis.

The better immune reconstitution observed in LTHIs is consistent with previous evidence showing that incomplete immune recovery after ART is associated with adverse clinical outcomes. Kelly et al. reported in a systematic review that discordant immune response under ART was linked to increased mortality and a higher frequency of clinical events (7). Similarly, among patients with advanced HIV disease starting their first ART regimen, poorer immunovirological recovery has been associated with less favorable outcomes, although these outcomes may also be influenced by regimen choice and baseline disease severity (23). Earlier studies of discordant virological and immunological responses likewise showed poorer clinical outcomes than those observed in full responders (24, 25), and poor early CD4 recovery despite viral suppression has also been linked to subsequent AIDS or death (26). The present study is broadly consistent with these findings.

The long-term survival results further support the clinical importance of these differences. Kaplan–Meier curves showed a clear and persistent separation between the two groups, and Cox regression indicated that LTHIs had a lower risk of all-cause mortality than TPs. This pattern is generally in line with previous HIV cohort studies. May et al. (27) demonstrated in a large collaborative cohort that lower CD4 counts at ART initiation were associated with higher mortality, particularly during the earlier years after ART initiation. Engsig et al. (10) further showed that even among individuals with sustained virological suppression for more than 3 years, persistently poor CD4 recovery remained associated with significantly increased mortality. More recently, Castillo-Rozas et al. (28) suggested that immune recovery during follow-up may be more strongly associated with mortality than baseline CD4 alone. Even under sustained viral suppression, the risk of AIDS or death continues to follow a CD4 gradient (29). In the PISCIS cohort, immune recovery 2 years after ART initiation among late presenters was also strongly associated with long-term survival (30). Late presentation itself has likewise been associated with excess short-, mid-, and long-term mortality in national cohort analyses (31). Taken together, these studies support the interpretation that immune restoration after treatment initiation is not only a marker of the immunological response, but also a clinically relevant indicator of subsequent prognosis.

Several possible explanations may account for the poorer immune reconstitution and survival outcomes observed in TPs. First, TPs may have experienced more severe immune damage before or around ART initiation. TPs developed AIDS-related symptoms or signs within the average latency period, suggesting more rapid disease activity, deeper CD4^+^ T-cell depletion, persistent immune activation, and greater disruption of immune homeostasis. Persistent HIV-related immune activation and inflammation may continue despite ART and have been considered important contributors to CD4^+^ T-cell depletion and non-AIDS-related adverse outcomes (32). Poor immune reconstitution is also a multifactorial process involving several host-, immune-, and treatment-related mechanisms (33). At the cellular and molecular levels, incomplete immune recovery has been associated with abnormalities in CD4^+^ T-cell quantity and quality, other immune-cell populations, soluble molecules, and cytokine profiles (34). Such immune injury may not be fully reversible even after long-term virological suppression under ART, thereby limiting subsequent CD4^+^ T-cell recovery and contributing to worse clinical outcomes. This interpretation is consistent with previous evidence showing that lower CD4^+^ T-cell counts at ART initiation, incomplete CD4 recovery during treatment, and late presentation are associated with increased long-term mortality (10, 27, 28).

Second, the timing of treatment initiation may also have contributed to the observed group differences. Individuals who start ART at a more advanced stage of infection generally have less preserved immune reserve and may be less likely to achieve complete immune restoration, even if viral replication is subsequently controlled. Previous cohort studies have shown that late presentation and delayed immune recovery after ART initiation are strongly associated with poorer long-term survival (30, 31). In the present study, TPs may therefore represent a group in whom treatment was initiated after more substantial immunological and clinical deterioration had already occurred.

Third, baseline disease status may reflect an underlying difference in clinical vulnerability between the two groups. More advanced WHO clinical stage, higher viral burden, prior AIDS-related manifestations, and possible opportunistic conditions before treatment may all indicate a more severe disease phenotype in TPs. In contrast, LTHIs may represent a relatively stable treated phenotype characterized by slower clinical progression, better preservation of immune function, and more favorable long-term recovery. Previous studies have similarly shown that age, baseline CD4^+^ T-cell count, route of infection, and treatment-related factors are associated with progression to AIDS or death (21–23, 35). Therefore, the observed differences between LTHIs and TPs should not be interpreted simply as the direct effect of the group label itself, but rather as the long-term consequence of different disease progression patterns before and during ART care.

This study has several limitations. First, although propensity score matching was used to balance measured baseline characteristics between LTHIs and TPs, the observational design cannot fully exclude residual confounding. To reduce confounding by measured factors, we applied propensity score matching using key demographic, epidemiological, treatment-related, and baseline virological variables, and covariate balance was assessed using standardized mean differences. In the outcome analyses, regression models were further used to estimate effect sizes after matching. Nevertheless, some unmeasured factors, including comorbidities, coinfections, socioeconomic status, health-care access, substance use, and other behavioral factors, may still have influenced both immune recovery and long-term survival. Second, immune reconstitution was defined using the most recent CD4^+^ T-cell count before the data cut-off. This definition was chosen because it reflects the latest available immune status in routine cohort follow-up and allows a clinically interpretable comparison between groups. However, it does not capture the full longitudinal CD4^+^ T-cell trajectory, the speed of immune recovery, or transient fluctuations related to intercurrent illness, laboratory timing, or short-term clinical changes. Future studies using repeated CD4^+^ T-cell measurements and trajectory-based models are needed to provide a more detailed understanding of immune recovery patterns. Third, treatment adherence, ART regimen changes, treatment interruptions, and drug-related adverse effects were not fully evaluated in the present analysis. We attempted to reduce related bias by restricting the analysis to individuals with a history of ART and by including treatment-related variables available in the cohort, such as ART start year or ART duration, in the matching or descriptive analyses. However, more detailed regimen- and adherence-level information would be needed to fully assess their influence on immune restoration and mortality. Fourth, information on specific causes of death was limited; therefore, only all-cause mortality was analyzed. To improve the robustness of the survival analysis, we used Kaplan–Meier curves, log-rank tests, Cox proportional hazards models with robust standard errors clustered by matched pairs, and additional model checking for the proportional hazards assumption. In addition, the survival analysis further excluded participants without valid survival-related information or without positive follow-up time after phenotype classification. Because the number of excluded participants differed between LTHIs and TPs, differential exclusion may have affected covariate balance in the survival-analysis subset. Although most key matching covariates remained balanced, mild residual imbalance was observed for ART start year and baseline VL category, and potential selection bias could not be fully excluded. Finally, this study was conducted in a single regional cohort in Xinjiang, China. The standardized cohort management system and long-term follow-up strengthened internal consistency, but the generalizability of the findings to other populations and health-care settings requires further validation.

## 5 Conclusion

Overall, the present study shows that, even under long-term ART, LTHIs and TPs differ consistently in both immune reconstitution and long-term survival. Compared with LTHIs, TPs were less likely to achieve adequate immune recovery and more likely to experience all-cause mortality during follow-up. Although the survival advantage of LTHIs decreased over time, it remained evident throughout follow-up. These findings suggest that disease progression patterns reflect not only past clinical course but also subsequent immune recovery and long-term prognosis. Further studies with larger samples and longer follow-up are needed to validate this framework and to clarify its value for individualized long-term HIV management.

## Data Availability

The original database contains confidential patient information and is not publicly available to protect participant privacy. The anonymized data supporting the conclusions of this article will be made available by the corresponding author upon reasonable request, subject to applicable ethical and privacy restrictions.

## References

[1] INSIGHT START Study Group Lundgren JD Babiker AG Gordin F Emery S Grund B . Initiation of antiretroviral therapy in early asymptomatic HIV infection. N Engl J Med. (2015) 373:795–807. doi: 10.1056/NEJMoa1506816, 26192873 PMC4569751

[2] TEMPRANO ANRS 12136 Study Group Danel C Moh R Gabillard D Badje A Le Carrou J . A trial of early antiretrovirals and isoniazid preventive therapy in Africa. N Engl J Med. (2015) 373:808–22. doi: 10.1056/NEJMoa1507198 26193126

[3] Zhao Y Wu Z McGoogan JM Shi CX Li A Dou Z . Immediate antiretroviral therapy decreases mortality among patients with high CD4 counts in China: a nationwide, retrospective cohort study. Clin Infect Dis. (2018) 66:727–34. doi: 10.1093/cid/cix878, 29069362 PMC5850406

[4] Deeks SG Lewin SR Havlir DV. The end of AIDS: HIV infection as a chronic disease. Lancet. (2013) 382:1525–33. doi: 10.1016/S0140-6736(13)61809-7, 24152939 PMC4058441

[5] Yang X Su B Zhang X Liu Y Wu H Zhang T. Incomplete immune reconstitution in HIV/AIDS patients on antiretroviral therapy: challenges of immunological non-responders. J Leukoc Biol. (2020) 107:597–612. doi: 10.1002/JLB.4MR1019-189R, 31965635 PMC7187275

[6] Chen C Chen H Wu L Gong Q He J. Factors influencing rapid antiretroviral therapy initiation in Jiulongpo, Chongqing, China: a retrospective cohort from 2018 to 2022. AIDS Res Ther. (2024) 21:15. doi: 10.1186/s12981-024-00601-y, 38494484 PMC10944594

[7] Kelly C Gaskell KM Richardson M Klein N Garner P MacPherson P. Discordant immune response with antiretroviral therapy in HIV-1: a systematic review of clinical outcomes. PLoS One. (2016) 11:e0156099. doi: 10.1371/journal.pone.0156099, 27284683 PMC4902248

[8] Vos WAJW Navas A Meeder EMG Blaauw MJT Groenendijk AL van Eekeren LE . HIV immunological non-responders are characterized by extensive immunosenescence and impaired lymphocyte cytokine production capacity. Front Immunol. (2024) 15:1350065. doi: 10.3389/fimmu.2024.1350065, 38779686 PMC11109418

[9] Engsig FN Gerstoft J Kronborg G Larsen CS Pedersen G Røge B . Long-term mortality in HIV patients virally suppressed for more than three years with incomplete CD4 recovery: a cohort study. BMC Infect Dis. (2010) 10:318. doi: 10.1186/1471-2334-10-318, 21044307 PMC2988053

[10] Engsig FN Zangerle R Katsarou O Dabis F Reiss P Gill J . Long-term mortality in HIV-positive individuals virally suppressed for >3 years with incomplete CD4 recovery. Clin Infect Dis. (2014) 58:1312–21. doi: 10.1093/cid/ciu038, 24457342 PMC6276895

[11] Mussini C Lorenzini P Cozzi-Lepri A Lapadula G Marchetti G Nicastri E . CD4/CD8 ratio normalisation and non-AIDS-related events in individuals with HIV who achieve viral load suppression with antiretroviral therapy: an observational cohort study. Lancet HIV. (2015) 2:e98–e106. doi: 10.1016/S2352-3018(15)00006-5, 26424550

[12] Han WM Apornpong T Kerr SJ Hiransuthikul A Gatechompol S Do T . CD4/CD8 ratio normalization rates and low ratio as prognostic marker for non-AIDS defining events among long-term virologically suppressed people living with HIV. AIDS Res Ther. (2018) 15:13. doi: 10.1186/s12981-018-0200-4, 30261902 PMC6158807

[13] Serrano-Villar S Pérez-Elías MJ Dronda F Casado JL Moreno A Royuela A . Increased risk of serious non-AIDS-related events in HIV-infected subjects on antiretroviral therapy associated with a low CD4/CD8 ratio. PLoS One. (2014) 9:e85798. doi: 10.1371/journal.pone.0085798, 24497929 PMC3907380

[14] Marcus JL Leyden WA Alexeeff SE Anderson AN Hechter RC Hu H . Comparison of overall and comorbidity-free life expectancy between insured adults with and without HIV infection, 2000–2016. JAMA Netw Open. (2020) 3:e207954. doi: 10.1001/jamanetworkopen.2020.7954, 32539152 PMC7296391

[15] Kumar P. Long term non-progressor (LTNP) HIV infection. Indian J Med Res. (2013) 138:291–3. 24135172 PMC3818590

[16] Buchbinder S Vittinghoff E. HIV-infected long-term nonprogressors: epidemiology, mechanisms of delayed progression, and clinical and research implications. Microbes Infect. (1999) 1:1113–20. doi: 10.1016/S1286-4579(99)00204-X, 10572315

[17] Pantaleo G Menzo S Vaccarezza M Graziosi C Cohen OJ Demarest JF . Studies in subjects with long-term nonprogressive human immunodeficiency virus infection. N Engl J Med. (1995) 332:209–16. doi: 10.1056/NEJM199501263320402, 7808486

[18] Kennedy BD Blazkova J Justement JS Shi V Rai MA Manning MR . Comprehensive analysis of HIV reservoirs in elite controllers. J Clin Invest. (2023) 133:e165446. doi: 10.1172/JCI165446, 36719383 PMC9888367

[19] Han J Wu Z McGoogan JM Mao Y Tang H Li J . Overrepresentation of injection drug use route of infection among human immunodeficiency virus long-term nonprogressors: a nationwide, retrospective cohort study in China, 1989–2016. Open Forum Infect Dis. (2019) 6:ofz182. doi: 10.1093/ofid/ofz182, 31139671 PMC6527089

[20] Li Y Ni Y He Q Hu X Zhang Y He X . Survival analysis and immune differences of HIV long-term non-progressors in Xinjiang China: a 12-year prospective cohort observation. AIDS Behav. (2024) 28:3151–60. doi: 10.1007/s10461-024-04396-x, 38869754 PMC11390762

[21] Jiang H Xie N Cao B Tan L Fan Y Zhang F . Determinants of progression to AIDS and death following HIV diagnosis: a retrospective cohort study in Wuhan, China. PLoS One. (2013) 8:e83078. doi: 10.1371/journal.pone.0083078, 24376638 PMC3871665

[22] Luo H Sun M Du J. Associated factors for progression to AIDS among HIV-infected people who use drugs: a retrospective cohort study in Dongguan, China. BMJ Open. (2019) 9:e023841. doi: 10.1136/bmjopen-2018-023841, 31272970 PMC6615836

[23] Burgos J Moreno-Fornés S Reyes-Urueña J Bruguera A Martín-Iguacel R Raventos B . Mortality and immunovirological outcomes in patients with advanced HIV disease on their first antiretroviral treatment: differential impact of antiretroviral regimens. J Antimicrob Chemother. (2022) 78:108–16. doi: 10.1093/jac/dkac361, 36308326

[24] Piketty C Weiss L Thomas F Mohamed AS Belec L Kazatchkine MD. Long-term clinical outcome of human immunodeficiency virus-infected patients with discordant immunologic and virologic responses to a protease inhibitor-containing regimen. J Infect Dis. (2001) 183:1328–35. doi: 10.1086/319861, 11294663

[25] Zoufaly A an der Heiden M Kollan C Bogner JR Fätkenheuer G Wasmuth JC . Clinical outcome of HIV-infected patients with discordant virological and immunological response to antiretroviral therapy. J Infect Dis. (2011) 203:364–71. doi: 10.1093/jinfdis/jiq055, 21208929 PMC3130441

[26] Takuva S Maskew M Brennan AT Long L Sanne I Fox MP. Poor CD4 recovery and risk of subsequent progression to AIDS or death despite viral suppression in a south African cohort. J Int AIDS Soc. (2014) 17:18651. doi: 10.7448/IAS.17.1.18651, 24594114 PMC3942566

[27] May MT Vehreschild JJ Trickey A Obel N Reiss P Bonnet F . Mortality according to CD4 count at start of combination antiretroviral therapy among HIV-infected patients followed for up to 15 years after start of treatment: collaborative cohort study. Clin Infect Dis. (2016) 62:1571–7. doi: 10.1093/cid/ciw183, 27025828 PMC4885653

[28] Castillo-Rozas G Tu S Luz PM Mejia F Sierra-Madero J Rouzier V . Clinical outcomes and risk factors for immune recovery and all-cause mortality in Latin Americans living with HIV with virological success: a retrospective cohort study. J Int AIDS Soc. (2024) 27:e26214. doi: 10.1002/jia2.26214, 38494667 PMC10945036

[29] Opportunistic Infections Project Team of the Collaboration of Observational HIV Epidemiological Research in Europe (COHERE) in EuroCoord Young J Psichogiou M Meyer L Ayayi S Grabar S . CD4 cell count and the risk of AIDS or death in HIV-infected adults on combination antiretroviral therapy with a suppressed viral load: a longitudinal cohort study from COHERE. PLoS Med. (2012) 9:e1001194. doi: 10.1371/journal.pmed.1001194, 22448150 PMC3308938

[30] Martin-Iguacel R Reyes-Urueña J Bruguera A Aceitón J Díaz Y Moreno-Fornés S . Determinants of long-term survival in late HIV presenters: the prospective PISCIS cohort study. EClinicalMedicine. (2022) 52:101600. doi: 10.1016/j.eclinm.2022.101600, 35958520 PMC9358427

[31] Sobrino-Vegas P Moreno S Rubio R Viciana P Bernardino JI Blanco JR . Impact of late presentation of HIV infection on short-, mid- and long-term mortality and causes of death in a multicenter national cohort: 2004–2013. J Infect. (2016) 72:587–96. doi: 10.1016/j.jinf.2016.01.017, 26920789

[32] Lv T Cao W Li T. HIV-related immune activation and inflammation: current understanding and strategies. J Immunol Res. (2021) 2021:7316456. doi: 10.1155/2021/7316456, 34631899 PMC8494587

[33] Zhang W Ruan L. Recent advances in poor HIV immune reconstitution: what will the future look like? Front Microbiol. (2023) 14:1236460. doi: 10.3389/fmicb.2023.1236460, 37608956 PMC10440441

[34] Yan L Xu K Xiao Q Tuo L Luo T Wang S . Cellular and molecular insights into incomplete immune recovery in HIV/AIDS patients. Front Immunol. (2023) 14:1152951. doi: 10.3389/fimmu.2023.1152951, 37205108 PMC10185893

[35] Choi Y Choi BY Kim SI Choi J Kim J Park BY . Effect of characteristics on the clinical course at the initiation of treatment for human immunodeficiency virus infection using dimensionality reduction. Sci Rep. (2023) 13:5547. doi: 10.1038/s41598-023-31916-x, 37016006 PMC10073208

